# ACE2, the Counter-Regulatory Renin–Angiotensin System Axis and COVID-19 Severity

**DOI:** 10.3390/jcm10173885

**Published:** 2021-08-29

**Authors:** Filippos Triposkiadis, Andrew Xanthopoulos, Grigorios Giamouzis, Konstantinos Dean Boudoulas, Randall C. Starling, John Skoularigis, Harisios Boudoulas, Efstathios Iliodromitis

**Affiliations:** 1 Department of Cardiology, Larissa University General Hospital, 41110 Larissa, Greece; andrewvxanth@gmail.com (A.X.); grgiamouzis@gmail.com (G.G.); iskoular@gmail.com (J.S.); 2 Department of Medicine/Cardiovascular Medicine, The Ohio State University, Columbus, OH 43210, USA; Konstantinos.Boudoulas@osumc.edu (K.D.B.); boudoulas@bioacademy.gr (H.B.); 3 Kaufman Center for Heart Failure Treatment and Recovery, Heart, Vascular, and Thoracic Institute, Cleveland Clinic, Cleveland, OH 44195, USA; starlir@ccf.org; 4 Second Department of Cardiology, National and Kapodistrian University of Athens, Attikon University Hospital, 12462 Athens, Greece; iliodromitis@yahoo.gr

**Keywords:** COVID-19, ACE2, renin–angiotensin system

## Abstract

Angiotensin (ANG)-converting enzyme (ACE2) is an entry receptor of severe acute respiratory syndrome coronavirus 2 (SARS-CoV-2) that causes coronavirus disease 2019 (COVID-19). ACE2 also contributes to a deviation of the lung renin–angiotensin system (RAS) towards its counter-regulatory axis, thus transforming harmful ANG II to protective ANG (1–7). Based on this purported ACE2 double function, it has been put forward that the benefit from ACE2 upregulation with renin–angiotensin–aldosterone system inhibitors (RAASi) counterbalances COVID-19 risks due to counter-regulatory RAS axis amplification. In this manuscript we discuss the relationship between ACE2 expression and function in the lungs and other organs and COVID-19 severity. Recent data suggested that the involvement of ACE2 in the lung counter-regulatory RAS axis is limited. In this setting, an augmentation of ACE2 expression and/or a dissociation of ACE2 from the ANG (1–7)/Mas pathways that leaves unopposed the ACE2 function, the SARS-CoV-2 entry receptor, predisposes to more severe disease and it appears to often occur in the relevant risk factors. Further, the effect of RAASi on ACE2 expression and on COVID-19 severity and the overall clinical implications are discussed.

## 1. Introduction

The rapid spread of severe acute respiratory syndrome coronavirus 2 (SARS-CoV-2) has led to a sudden outbreak of coronavirus disease 2019 (COVID-19) that has affected the entire world. COVID-19 clinical manifestations range from asymptomatic infection to severe disease, with pneumonia and acute respiratory distress syndrome (ARDS) set in motion by inflammation, large-vessel thrombosis, and in situ microthrombi [1].

SARS-CoV-2, which is usually transmitted by respiratory particles released from an infected subject, initially targets the nasal multiciliated epithelial cells [2]. Subsequently, the infection spreads to the upper airways and in selected patients to the deepest parts of the lungs, where it targets the type II pneumocytes causing severe pneumonia and acute respiratory distress syndrome (ARDS) [3]. Finally, massive systemic multiorgan infection may occur, as the virus targets the epithelial cells and the endothelial cells of the capillaries [4], the two main components of tissue barriers which are of utmost importance for health [5]. The infection and damage of cells of tissue barriers allow the virus entrance to the bloodstream and lymphatic system, spreading to several organs including the heart [6], the kidney [7], and the brain [8,9]. This mechanism may also explain why pulmonary infection can occur after functional exclusion of the upper airways from the lungs (e.g., after total laryngectomy) [10]. It should be noted, however, that viremia is not the main route of SARS-CoV-2 spreading [11].

The SARS-CoV-2 enters the host cells through binding to the angiotensin-converting enzyme (ACE2) receptor after activation of the S1 domain of SARS-CoV-2 spike (S) protein by an ACE2 co-factor, the transmembrane protease serine 2 (TMPRSS2) [12,13]. The ACE2 receptor, however, is an important member of the counter-regulatory axis of the renin–angiotensin system (RAS), which parallel to its pivotal function in fluid volume control, plays a major role for other functions as well, including stem cell maintenance and differentiation, hematopoiesis, vasculogenesis, erythropoiesis, myeloid differentiation, inflammation, and innate and adaptive immunity, among others [14,15].

It has been posited that down-regulation of membrane-bound ACE2 by SARS-CoV-2 eliminates the function of the counter-regulatory RAS axis, that in turn escalates the severity of the inflammation observed in SARS-CoV-2 [16,17]. It has been suggested that the renin–angiotensin–aldosterone system (RAAS) inhibitors (RAASi), including ACE inhibitors (ACEi), angiotensin II (ANG II) receptor blockers (ARB), and mineralocorticoid receptor antagonists (MRA), increase ACE2 receptor expression [18,19,20], and thus, the benefits from the anti-inflammatory effects originating from upregulation of the counter-regulatory RAS axis may counterbalance the risks when using RAASi in the COVID-19 era [21].

In this manuscript we discuss the relationship between ACE2 expression and function in the lungs and other organs and COVID-19 severity. Recent data suggested that the involvement of ACE2 in the lung counter-regulatory RAS axis is limited. In this setting, an augmentation of ACE2 expression and/or a dissociation of ACE2 from the ANG (1–7)/Mas pathways that leaves unopposed the ACE2 function, the SARS-CoV-2 entry receptor, predisposes to more severe disease and it appears to often occur in the relevant risk factors. Further, the effect of RAASi on ACE2 expression and on COVID-19 severity and the overall clinical implications are discussed.

## 2. The Lung Counter-Regulatory RAS AXIS and COVID-19

According to the classic view, RAS is a sequence of assorted enzymatic steps that build up in the production of a single biologically active metabolite, the octapeptide angiotensin ANG II, by ACE [22]. However, new roles for certain intermediate products have been disclosed [23]; they may be processed in different ways by various enzymes, the most well-known being the ACE homolog ACE2. One effect is to set up a second counter-regulatory axis through ACE2/ANG (1–7), whose end-point metabolite ANG (1–7) occupies MAS receptors. ACE2 and other enzymes can generate ANG (1–7) directly or indirectly from either the decapeptide ANG I or from ANG II that acts on the receptor MAS to regulate multiple mechanisms in the heart, kidney, brain, and other tissues. In many cases, this counter-regulatory axis appears to compensate for or adjust the effects of the classical axis by mediating protective effects including vasodilation, improvement of endothelial function, inhibition of smooth muscle cell hypertrophy and migration, as well as inhibition of inflammation and thrombosis [23]. However, this may not occur in the lungs, and for this reason an augmentation of membranous ACE2 expression cannot be a therapeutic option, as is discussed in the following sections.

### 2.1. The SARS-CoV-2 Induced ACE2 Downregulation Is of Doubtful Relevance

**Figure 1:**
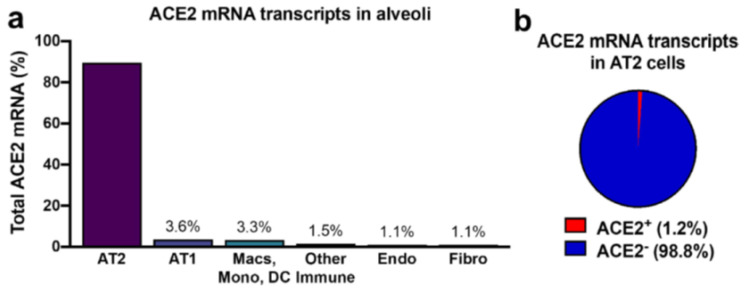
Angiotensin-converting enzyme (ACE)2 expression in human lung. (**a**) Approximately 89.5% of the cells with detectable ACE2 mRNA in the alveoli are alveolar type II cells. (**b**) Only 1–2% of alveolar type II cells have ACE2 mRNA transcripts. Abbreviations: AT2, alveolar type II; AT1, alveolar type I; Macs, Macrophages; Mono, Monocytes; DC, dendritic cells.; Other immune cells, B cells, mast cells, natural killer/T cells; Endo; Endothelial; Fibro, Fibroblasts/myofibroblasts [27].

It has been surmised that the harmful effects of SARS-CoV-2 are due to ACE2 downregulation that takes place after virus entrance into the cell by endocytosis [24]. However, various studies employing miscellaneous technologies demonstrated that ACE2 expression in the lungs is low. Hikmet and colleagues assessed the expression pattern of ACE2 covering > 150 different cell types commensurate to all major human tissues and organs [25]. ACE2 expression was for the most part observed in enterocytes, renal tubules, gallbladder, cardiomyocytes, male reproductive cells, placental trophoblasts, ductal cells, eye, and vasculature, whereas in the respiratory system, the ACE2 expression was finite [25]. In another study, the ACE2:ACE ratio in the lungs was 1:20, whereas in kidneys the ACE2:ACE ratio was roughly 1:1 [26]. In the respiratory system, ACE2 protein is abundant within regions of the sinonasal cavity, whereas in the lung parenchyma, ACE2 protein is located in a small subset of alveolar type II cells colocalized with TMPRSS2, a cofactor for SARS-CoV2 entry (Figure 1) [27,28]. Ultimately, ACE2 expression levels are low in the lung AT2, being 4.7-fold lower than the average expression level of all ACE2 expressing cell types [29]. 

Considering that SARS-CoV-2 and other viruses that enter via the lung use ACE2 as receptors, the low levels of ACE2 in the lung might help to restrict entry and impart an evolutionary gain in survival. This assumption is further supported by experimental studies demonstrating that lineages of transgenic (Tg) mice expressing high human ACE2 (hACE2; AC70 mice) demonstrated significantly higher lethality post-SARS-CoV infection than the lineages with low hACE2 expression (AC22 mice) [30,31]. Viral replication in the lungs reached a maximum at day 1 post-infection (p.i.), in which averages of 10^8.5^ and 10^8.7^ tissue culture infective doses (TCID_50_) SARS-CoV/gram were recovered from AC70 and AC22 mice, respectively, and gradually declined thereafter. However, at day 5 p.i. a significantly higher level of viral replication was sustained in the lungs of a single AC70 mouse than any AC22 mice [31].

### 2.2. The Binding of SARS-CoV-2 with ACE2 May Ignite a Catastrophic Inflammatory Response

SARS-CoV-2 induced cell destruction triggers a local immune response characterized by recruitment of macrophages and monocytes that release cytokines and prime adaptive T and B cell immune responses contributing in most cases to the final elimination of the pathogen [32,33]. However, in certain instances, especially if risk factors (e.g, old age, smoking, pollutants, other) are involved, there is inefficacious control of viral replication, viral propagation and eventually infection of the lower airways. Viral escape is accompanied by a huge release of pro-inflammatory cytokines (sepsis-like inflammation or cytokine storm) due to hyperactivation of the innate immune system and along with the inhibition of the adaptive immune response may result in severe disease and/or death [34]. 

In autopsy of patients who succumbed to severe acute respiratory syndrome, SARS-CoV S protein and its RNA were only detected in ACE2^+^ cells in the lungs and other organs, and high levels of proinflammatory cytokines were exclusively detected in the SARS-CoV-infected ACE2^+^ cells [35]. Expression of ACE2 is tightly linked to innate and acquired immune responses, regulation of B cell-mediated immunity, and cytokine secretion, indicating that an elevated expression of ACE2 may lengthen the virus life cycle, intensify virus replication, and bring about entry of the virus into the host cell [36,37]. Taken together, occupation of ACE2 by SARS-Cov-2 may ignite inflammatory signaling, and any intervention elevating lung ACE2 expression may amplify this process.

### 2.3. Prolyloligopeptidase (POP), Also Named Prolylendopeptidase, Rather Than ACE2 Seems to Underlie ANG (1–7) Generation in the Lungs

A recent ex vivo study examined the partial contribution to ANG (1–7) generation from ANG II by ACE2 and POP in serum, kidney, and lung tissues. POP, and not ACE2, was the principal enzyme responsible for ANG II transformation to ANG (1–7) in the circulation and the lungs [38]. In the same study, it was also demonstrated that POP is significantly less effective in transforming the harmful ANG II to the protective ANG (1–7) compared with ACE2. 

POP is a cytoplasmic enzyme, but its activity can also be quantified in body fluids. Peptides up to 30 amino acids long that contain a proline are potential substrates of POP. Many years of experimental work have indicated that POP has not a single physiologically distinguishing role but several roles depending on the milieu in which POP is located [39]. Moreover, POP is proinflammatory as it concurrently participates in the generation of the matrikine proline-glycine-proline (PGP) from collagen fragments; (PGP) has traditionally been characterized as a neutrophil chemoattractant [40]. The commencing cleavage of native collagen by matrix metalloproteinases (MMPs) originates from a variety of cellular elements producing appropriately sized substrate fragments for POP that subsequently act to free PGP followed by acetylation and transformation to acetyl PGP (AcPGP), which is 4–7-fold more potent. 

### 2.4. ACE2 Contributes to SARS-CoV-2 Dissemination within the Organism

Flow cytometry studies have demonstrated marked upregulation of ACE2 expression on the activated alveolar macrophages (significantly higher in the inflammatory M1 than in the anti-inflammatory M2 macrophages), with little to no expression of ACE2 on most of the human peripheral blood-derived immune cells (e.g., CD4+ T, CD8+ T, activated CD4+/CD8+ T, Tregs, Th17, NKT, B, NK cells, monocytes, dendritic cells, and granulocytes) [41]. Accordingly, alveolar macrophages, by virtue of their polarization state toward either an M1 or M2 phenotype, function in a different way following SARS-CoV-2 infection. M1 alveolar macrophages are hijacked by SARS-CoV-2, allowing the viral infection and spread, whereas M2 alveolar macrophages degrade the virus and limit its spread [42]. Thus, while macrophages play an important role in antiviral defense mechanisms, in the case of SARS-CoV-2 due to ACE2 overexpression in the M1 type, they may also serve as a Trojan horse by enabling pulmonary SARS-CoV-2 invasion, facilitating engraftment, producing prolonged local and systemic uncontrolled inflammatory responses, and therefore governing the severity of infection [43].

**Figure 2:**
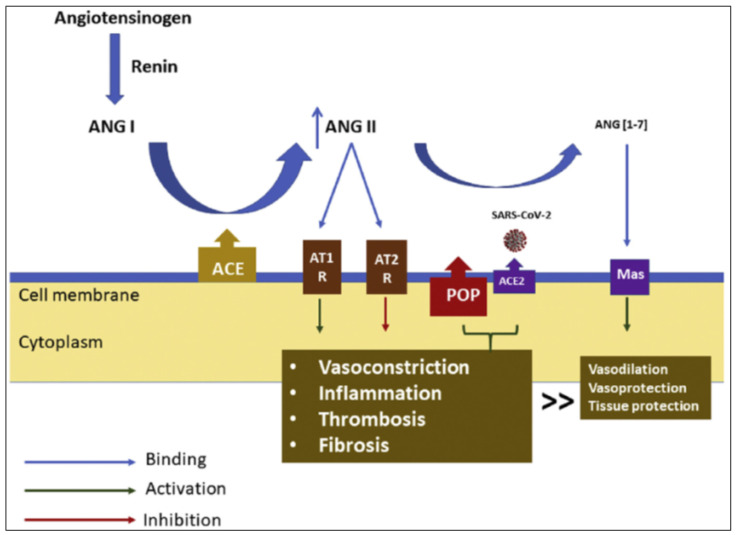
The counter-regulatory renin–angiotensin system (RAS) axis in the lungs in the coronavirus disease 2019 (COVID-19) setting. Angiotensin-converting enzyme 2 (ACE2) serves as the severe acute respiratory syndrome coronavirus (SARS-CoV)-2 receptor, whereas the proinflammatory prolyloligopeptidase (POP) converts less effectively than ACE2 the deleterious angiotensin (ANG) II to the protective ANG (1–7). As a result, deleterious signalling (inflammation, thrombosis and fibrosis) dominates over protective (vasodilation, vasoprotection and tissue protection) signalling in patients with severe COVID-19, which may lead to acute lung injury. Abbreviations: AT1R, angiotensin receptor type 1; AT2R, angiotensin receptor type 2 [45].

Thus, from a (patho)physiological point-of-view an increase in the membrane bound ACE2, the SARS-CoV-2 receptor, in the lungs will most likely increase susceptibility to COVID-19 [44], as it is dubious whether it will bolster the counter-regulatory RAS axis (Figure 2) [38,45]. 

## 3. ACE2 Expression and the Counter-Regulatory RAS AXIS in States Predisposed to Severe COVID-19

There is compelling evidence that cancer, chronic obstructive pulmonary disease (COPD), chronic kidney disease (CKD), heart disease, obesity (body mass index (BMI) ≥ 30 kg/m^2^), type 2 diabetes mellitus (T2DM) and solid organ transplantation place adults of any age at increased risk of severe illness from SARS-CoV-2 [46].

### 3.1. Cancer

In cancer patients with COVID-19, all-cause mortality is high and related to general and cancer-specific risk factors [47]. Some of the general risk factors include age, male sex, number of comorbidities, cardiopulmonary disease, and smoking status, whereas cancer-specific features that have been identified as being associated with worse outcomes include tumour stage, disease progression, and type of cancer; some studies identify thoracic cancers as being associated with increased risk compared with other solid tumors [48]. 

The RAS is indispensable for stem cell maintenance and differentiation and plays a crucial role in tumorigenesis and cancer progression, suggesting that these roles may intersect and result in regulation of cancer stem cell (CSC) function by the RAS [15]. Elements of the RAS are highly expressed in many cancer types. ACE2 expression is raised in non-small cell lung cancer including adenocarcinoma and squamous cell carcinoma [49]. Moreover, a recent study reported that ACE2 and TMPRSS2 levels are higher at resection margins of lung cancer survivors than those in normal tissues of non-cancerous individuals [50].

Based on comprehensive promoter analysis of ACE-2, Gottschalk and colleagues proposed that STAT3-mediated upregulation of ACE2 might play a critical role both in COVID-19 infection and lung tumor progression [51]. In this regard, SARS-CoV-2 infection might stimulate the binding of STAT3 in the promoter of ACE2, resulting in enhanced expression of ACE-2, which in turns upscales virus entry through the ACE2 receptor. On the other hand, cancer pathologies in lung tissue also switch on the transcription of STAT3, leading to the upregulated expression of ACE2 in cancer cells [51].

ACE2 expression is also raised in renal cancer and gastrointestinal cancer [52]. It is noteworthy that ACE2 and TMPRSS2 are expressed at high levels on tumor and normal colorectal epithelial tissues and that patients with colorectal cancer and COVID-19 are more likely to have lymphopenia, higher respiratory rate, and high hypersensitive C-reactive protein levels than matched patients with COVID-19 but without cancer [53,54]. Finally, landscape profiling analyses on the expression level of ACE2 in pan-cancers have revealed that the risk for development of SARS-CoV-2 infection was coupled with the expression level of ACE2 [36]. 

### 3.2. Chronic Obstructive Pulmonary Disease

COPD is a significant risk factor for hospitalization, intensive care unit stay, and mortality in patients with COVID-19 [55,56].

COPD patients are usually cigarette smokers, though long-term exposure to other lung irritants, such as secondhand smoke, is also responsible for COPD development. Pulmonary ACE2 gene expression is upregulated in ever-smokers compared with nonsmokers irrespective of COPD status [57]. A meta-analysis demonstrated a 25% increase in pulmonary ACE2 expression in ever-smokers and a trend for higher ACE2 levels in COPD patients [57]. In another study ACE2 mRNA expression was significantly higher in the lung tissue of current smokers without airflow limitation and current smokers with COPD (GOLD stages II and III–IV) as compared with never-smokers. In addition, ex-smokers without airflow limitation had significantly lower ACE2 mRNA levels as compared with current smokers [58]. Leung and colleagues investigated gene expression levels of ACE-2 in the airways of individuals with and without COPD in three different cohorts and found that COPD and current smokers had significantly increased expression of ACE-2 [59]. Importantly, gene expression levels of ACE-2 were inversely related to an individual’s forced expiratory volume 1 (FEV1), suggesting a dose-dependent response. Lastly, a differential expression of ACE2 and TMPRSS2 in nasal and bronchial airways relative to age and disease status was reported. Children were found to have significantly lower expression of COVID-19 receptors in the upper and lower airways (nasal and bronchial). Moreover, the lung airway expression of both ACE2 and TMPRSS2 was found to be significantly upregulated in smokers compared with non-smokers, and in patients with COPD compared with healthy subjects [60]. Likewise, Fliesser and colleagues observed increased ACE2 and TMPRSS2 expression in lung tissue with a concomitant decrease in protective sACE2 in COPD patients [61].

### 3.3. Chronic Kidney Disease

CKD has come up not only as the most prevalent comorbidity carrying an increased risk for severe COVID-19, but also as the comorbidity that imparts the highest risk of severe COVID-19 [62].

ACE2 is predominantly expressed in the brush border of proximal tubular cells, less in the podocytes and vascular endothelial cells, and not at all in the glomerular endothelial and mesangial cells [63]. The functional role of ACE2 in the kidney has not been delineated. Although ACE2 has been described as a crucial player in the enzymatic conversion of ANG II to ANG (1–7), this may not be the case in the kidney, in which neprilysin (NEP) seems to be the major source of renal ANG (1–7) [64]. Indeed, it is estimated that in the healthy human kidneys, ACE2, prolylcarboxypeptidase (PCP), and POP together contribute less than 15% of total ANG (1–7) production [65].

**Figure 3:**
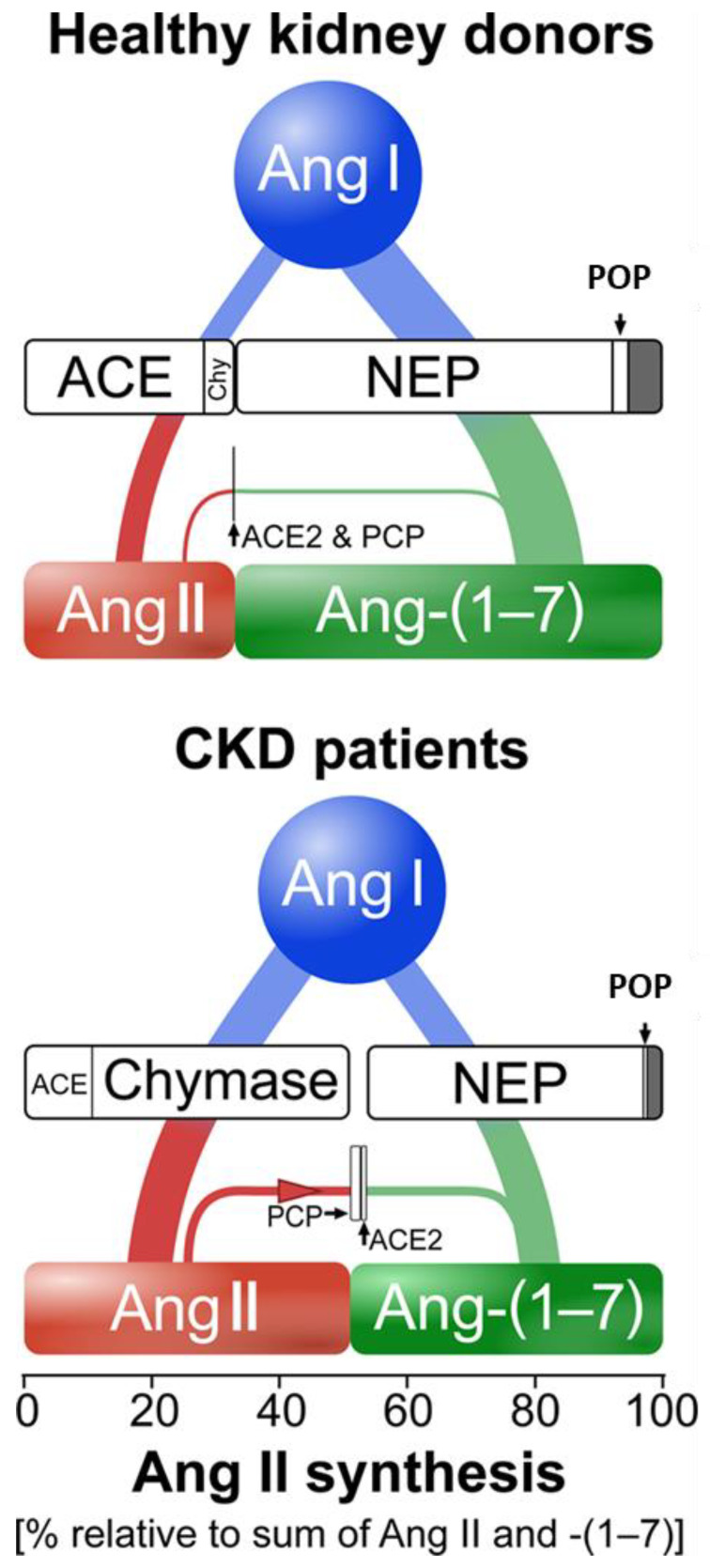
Overview of angiotensin processing by kidney-resident enzymes. Comparison of relative Ang (angiotensin) II and Ang (1–7) synthesis from Ang I in healthy kidney donors (top) and patients with chronic kidney disease (CKD; bottom). In healthy kidney donors, Ang I is mainly converted by NEP (neprilysin) to Ang (1–7) and to a smaller degree by ACE (angiotensin-converting enzyme) to Ang II. Minor contribution by chymase and POP (prolyl-endopeptidase) supplement Ang II and (1–7) synthesis, respectively. ACE2 and PCP (prolyl-carboxypeptidase)-mediated Ang II to Ang (1–7) conversion is present at a very low degree. Overall, the pathways producing Ang (1–7) exceed those which produce Ang II. B, In patients with CKD, Ang I is equally converted by chymase to Ang II and by NEP to Ang (1–7). Ang II synthesis is dominated by chymase and supplemented by ACE. POP-mediated Ang (1–7) synthesis is present at a very low degree. ACE2 and PCP-mediated Ang II to Ang (1–7) conversion is present at a higher degree than in healthy kidneys, albeit still minor in comparison to neprilysin’s activity. Thus, in comparison to healthy kidney donors, in patients with CKD severe renin–angiotensin system (RAS) dysregulation is exhibited, characterized by low Ang (1–7) production and high chymase-mediated Ang II production. In both cohorts, one or more unidentified enzymes contribute to a small portion of Ang I to Ang (1–7) conversion (depicted by gray area). The horizontal dimensions of the boxes equal median enzyme contribution [65].

There is severe RAS dysregulation in CKD [65]. In normal subjects, overall kidney ANG (1–7) generation exceeds ANG II generation by 2.6-fold and is 3.9 times higher than in CKD, indicating preponderance of the counter-regulatory RAS axis in healthy kidneys (Figure 3). In contrast, in CKD patients, chymase-dependent ANG II generation is 4.5 times higher than generation by ACE, which is compatible with serious RAS dysregulation. POP-mediated ANG (1–7) generation is extremely low. ACE2 and PCP-mediated ANG II to ANG (1–7) transformation is present at a higher level in CKD than in healthy kidneys, albeit minor compared with NEP activity. Collectively these findings suggest that both in normal subjects and patients with CKD the contribution of ACE2 to the renal counter-regulatory ANG (1–7)/Mas axis is limited. 

Tissue expression of ACE2 and TMPRSS2 were analyzed in renal tubulointerstitial and glomerular microarray expression data of healthy living donors (HLD) and patients with CKD obtained from the European Renal cDNA Bank. ACE2 expression was similar in the tubulointerstitium of the two groups, but lower in glomeruli of CKD patients compared to HLD. TMPRSS2 expression was similar in the tubulointerstitium but lower in glomeruli of CKD patients compared to HLD and there was a strong relationship between ACE2 and TMPRSS2 expression in the glomerulus [66]. Based on these findings it has been conjectured that the colocalization and co-expression of ACE2 and TMPRSS2 in the glomerulus and their strong correlation in this compartment may underlie the recent clinical observation of an emerging SARS-CoV-2-associated nephropathy (COVAN) [67].

### 3.4. Cardiac Disease

Many patients with COVID-19 suffer from underlying cardiac disease or develop acute cardiac injury during the course of the illness [68].

Cardiac ACE2 expression is highest in pericytes, but also detectable in vascular smooth muscle cells, fibroblasts, and cardiomyocytes [69]. In an experimental model of heart failure (HF), ACE2 immunoreactivity and mRNA levels increased in pulmonary, cardiac, and renal tissues in compensated but not in decompensated HF [70]. Elevated cardiac ACE2 expression at both mRNA and protein levels has also been reported in human HF [71]. Examination of the distribution of the cardiac ACE2 expression levels in dilated cardiomyopathy and hypertrophic cardiomyopathy has affirmed downregulation of ACE2 expression in fibroblasts, pericytes, and vascular smooth muscles with a concomitant upregulation of ACE2 expression in cardiomyocytes [72]. Similar findings have been reported in patients with aortic stenosis and HF with reduced ejection fraction [73].

A recent experimental study investigated the expression of several enzymes including ACE2 and TMPRSS2 in the lung, heart and kidneys of male Sprague Dawley rats with chronic HF created by a surgical aorto-caval fistula. Sham-operated rats served as controls [70]. ACE2 immunoreactivity and mRNA levels increased in pulmonary, cardiac and renal tissues of compensated but not of decompensated chronic HF. Interestingly, both the expression and abundance of pulmonary, cardiac and renal TMPRSS2 decreased in chronic HF in correlation with the severity of the disease. The authors conjectured that the increased expression of the ACE2 together with the suppression of TMPRSS2, which facilitates SARS-CoV-2 entry into ACE2, in HF may serve as a compensatory mechanism, counterbalancing the over-activity of the deleterious isoform, ACE [70].

### 3.5. Obesity

Obese individuals with COVID-19 are more likely to require hospital admission and intensive care unit stay and have increased mortality compared with healthy weight individuals [74]. 

Obesity is associated with an imbalance of the RAAS system [75]. Moreover, a recent experimental study found that the ACE2 expression was significantly higher in obese male mice relative to lean male controls or to obese female mice, whereas expression of TMPRSS2 in trachea was significantly lower in obese male mice relative to lean male controls and obese female mice. The authors proposed that these observations may potentially account, at least in part, for the association of obesity with SARS-CoV-2 infection and COVID-19 severity, as well as the male-biased mortality rate and that other cellular proteases may potentially contribute to SARS-CoV-2 entry into the TMPRSS2-negative cells [76]. 

ACE2 is broadly expressed in the adipose tissue and significantly more in the visceral than peripheral subcutaneous adipose tissue [77]. Consequently, obese individuals, especially those with visceral obesity, can acquire more viral load, which probably contributes to the increased COVID-19 severity compared with normal weight individuals [78]. Importantly, RAAS system imbalance reverses after weight loss [79].

### 3.6. Type 2 Diabetes Mellitus

T2DM is associated with poor outcomes after COVID-19 infection and with an increase in time required for viral clearance. T2DM is linked to raised ACE2 expression [80]. 

Experimental studies demonstrated that mice with diabetes have upregulated ACE2 and TMPRSS2 both in the lungs and the kidney [81]. In lung tissue samples, pulmonary ACE2 mRNA expression was similar between individuals with and without diabetes, whereas protein levels of ACE2 were significantly higher in both alveolar tissue and bronchial epithelium in individuals with diabetes; these findings were independent of smoking, COPD, BMI, RAASi use, and other potential confounders [82]. 

### 3.7. Solid Organ Transplantation

During the COVID-19 pandemic, concerns about adverse outcomes among organ transplant recipients, difficulties with infection prevention and control, and decreased resource availability led to a profound reduction in deceased donor organ transplantation, which was associated with a rise in waitlist deaths [83]. However, transplantation has currently returned to pre-2020 levels, reflecting the increasing understanding of the transplant community of the risks of deferring transplantation for patients on waiting lists [84]. 

Local RAS regulation is profoundly modified in solid organ transplant recipients. Heart transplant recipients are at a higher risk of SARS-COV-2 infection and have a twofold higher mortality in comparison to the general population if they acquire the disease [85]. Early after kidney transplantation (KTx), ANG II generation within the graft is ACE-mediated, while ANG (1–7) generation is dominantly mediated by NEP from ANG II. In aged allografts, a strong increase in local chymase-mediated ANG II synthesis associated with constant high NEP-mediated ANG (1–7) synthesis occurs [86]. Chymase expression may not only enhance local ANG II formation, but also restrict NEP/ANG (1–7)-mediated alternative RAS activation by exhausting ANG I as a substrate for NEP. Post-cardiac transplantation (HTx) patients with acute rejection show increased ACE activity but not ACE2, whereas those with chronic allograft vasculopathy show increased ACE2 activity [87].

However, despite the previously mentioned derangements, there is currently limited evidence supporting an independent relationship between the timing of transplant or recent induction immunosuppression and mortality from COVID-19. In general, the preponderance of evidence supports that age and coexisting morbidities, rather than immunosuppression, drive COVID-19 mortality among solid organ transplant recipients [84]. 

## 4. ACE2 and RAAS Inhibitors

Some early experimental studies demonstrated elevated expression of ACE2, the SARS-CoV-2 receptor, following the use of RAASi, raising concerns regarding the safety of these agents that currently form the backbone for the treatment of hypertension and HF in the COVID-19 era [18,20]. Current evidence regarding the potential of these agents to facilitate disease contraction and modify disease severity has been based on observational studies with the well-known significant limitations and on small, randomized control trials. 

The RAASi controversy during the COVID-19 outbreak was most likely unjustifiable after all, as recent data suggest that these drugs do not increase ACE2 expression. In the kidney cortex of mice receiving captopril or telmisartan there was no increase in ACE2 activity and protein compared with control mice [88]. In healthy young mice, neither the ACEi ramipril nor the ARB telmisartan affected lung or kidney ACE2 or TMPRSS2, except for a small increase in kidney ACE2 protein with ramipril [81]. In contrast, mice with comorbid diabetes had heightened lung ACE2 and TMPRSS2 protein levels and increased lung ACE2 activity. None of these parameters were affected by RAS blockade. ACE2 was similarly upregulated in the kidneys of mice with comorbid diabetes compared with aged controls, whereas TMPRSS2 (primarily distal nephron) was highest in telmisartan-treated animals [81].

**Figure 4:**
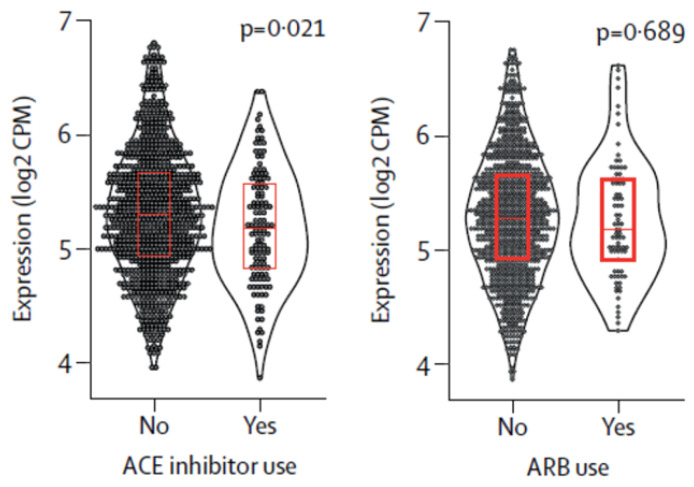
Effects of ACE inhibitor use, and ARB use on the expression of SARS-CoV-2 receptor (ACE2) in human lung tissue. Superimposed box plots show median (IQR). *p* values are from robust linear models, adjusted for current smoking status. ACE inhibitor use was associated with significantly lower ACE2 expression, whereas ARB was not. Abbreviations: ACE, angiotensin-converting enzyme; ARB, angiotensin II receptor blocker; CPM, counts per million; SARS-CoV-2, severe acute respiratory syndrome coronavirus 2 [89].

Examination of ACE2 gene expression in lung tissue samples revealed that ACEi and ARB did not enhance ACE2 expression (Figure 4) [89]. Likewise, there was no association between renal expression of ACE2 and either hypertension or common types of RAASi in kidney transcriptomes [90]; in another study, ACE2 expression was unaffected by either ACEi or ARB therapy [91]. In nasal cavity tissues and in the paranasal sinuses obtained both from healthy control donors and patients with chronic rhinosinusitis, the use of ACEi or ARB did not increase the expression of ciliary ACE2 receptors [92]. Finally, ACEi may increase ANG (1–7), the effector peptide product of ACE2, by inhibiting its degradation by ACE into angiotensin [1,2,3,4,5,93]. Regarding spironolactone, there is evidence to suggest that it may be protective in the COVID-19 setting by downregulating the androgen promoter of TMPRSS2 by its antiandrogenic actions and upregulating protease nexin 1 or serpin E2 (PN1), which in turn inhibits furin and plasmin, two of the processors of the spike protein [94]. Collectively, the above studies strongly support the notion that RAASi do not increase ACE2 expression and that the initial concerns regarding this issue were exaggerated. 

## 5. Clinical Implications

**Figure 5:**
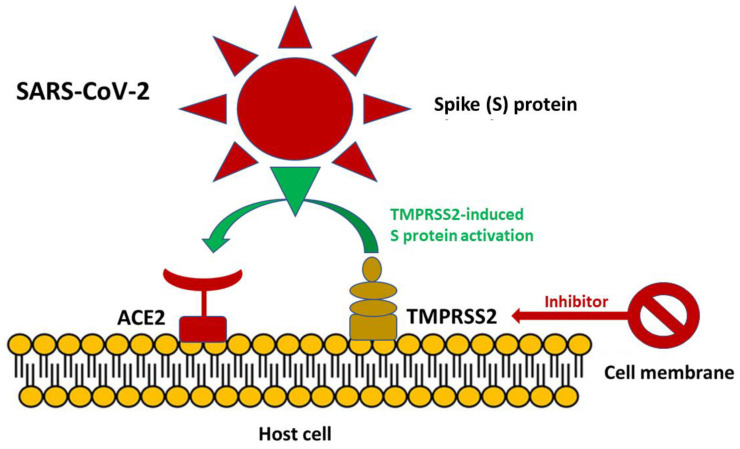
SARS-CoV-2 entry into ACE2 is facilitated by TMPRSS2 induced activation of the S protein via its serine protease activity. Inhibition of TMPRSS2 may furnish an alternative strategy to treat SARS-CoV-2 infection by blocking viral entry into host cells.

SARS-CoV-2 entry into ACE2, which is upregulated in many risk factors predisposing to severe COVID-19, is facilitated by TMPRSS2-induced activation of the S protein via its serine protease activity. This traditional observation suggests that the modulation of TMPRSS2 expression may furnish an alternative strategy to treat SARS-CoV-2 infection by blocking viral entry into host cells (Figure 5) [95]. The initial experimental data suggest that this treatment approach may be promising.

The hepatocyte growth factor activator inhibitor 2 (HAI-2) is a cognate inhibitor of TMPRSS2 [96]. In an experimental study expression of HAI-2 in human lung adenocarcinoma Calu-3 cells was knocked down (KD) by small interfering RNA transfection, and SARS-CoV-2 infection assays were performed. The level of viral RNA in HAI-2 KD cells was approximately 40 times greater than that in control cells, indicating that the endogenous level of HAI-2 in Calu-3 cells alleviated SARS-CoV-2 infection [97]. In accordance with the previous findings, some small molecules (e.g., homoharringtonine and halofuginone) that reduce surface expression of TMPRSS2 render cells exposed to them at drug concentrations known to be achievable in human plasma markedly resistant to SARS-CoV-2 infection in both live and pseudoviral in vitro models [98]. 

Based on the encouraging preliminary results, in a recent study a comprehensive structural modeling and binding-site analysis of the serine protease TMPRSS2 was performed, followed by a structure-based virtual screening against the National Center for Advancing Translational Sciences (NCATS) library consisting of up to 200,000 drug-like compounds designed from a diverse chemical space [99]. Selected hits were evaluated in the TMPRSS2 biochemical assay and the SARS-CoV-2-S pseudotyped particle entry assay, and a number of novel inhibitors were identified, providing a starting point for the further development of therapeutic drug candidates for COVID-19.

## 6. Conclusions

**Figure 6:**
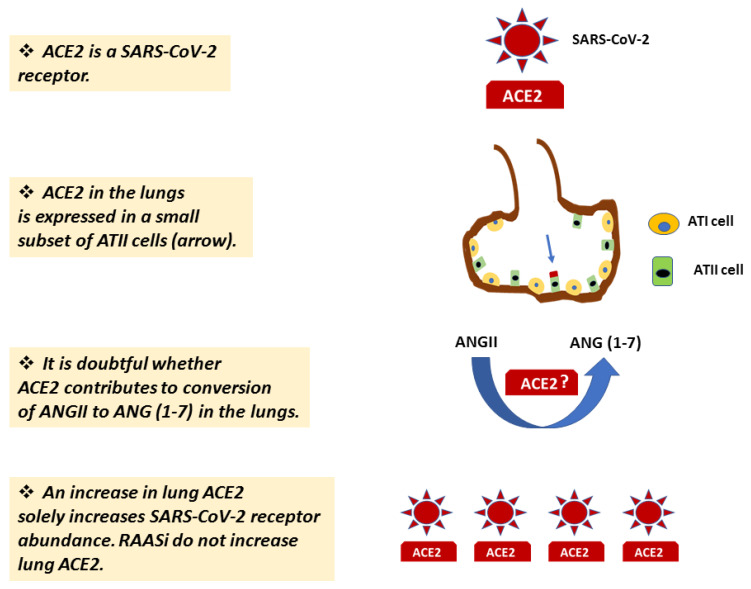
Expression and function of ACE2 in the lungs in the coronavirus disease 2019 (COVID-19) era. Abbreviations: ACE2, angiotensin-converting enzyme 2; SARS-CoV-2, severe acute respiratory distress syndrome coronary virus 2; ATI cell, lung alveolar epithelial type I cell; ATII cell, lung alveolar epithelial type II cell; ANG, angiotensin; RAASi, renin–angiotensin–aldosterone system inhibitors.

The SARS-CoV-2 enters ACE2, the main SARS-CoV-2 receptor, following primings of the viral S protein by the ACE2 cofactor TMPRSS2. ACE2 is additionally a crucial enzymatic player in several organs moving the RAS towards the counter-regulatory RAS axis by enzymatically transforming ANG II to ANG (1–7). However, this may not be the case in the lungs and kidneys, organs whose function is a major determinant of COVID-19 severity and outcome, where it predominantly serves as the SARS-CoV-2 receptor (Figure 6). Medical conditions and disease states associated with severe COVID-19 are characterized by an increase in ACE2 expression, dissociation of ACE2 from the ANG (1–7)/Mas pathway, or both. RAASi do not appear to affect COVID-19 severity by affecting lung ACE2 expression. The encouraging experimental results with agents that target TMPRSS2 should help expedite the rational design of human clinical trials designed to combat SARS-CoV-2 entry into ACE2 and active COVID-19 infection.

## Abbreviations

ACE: angiotensin-converting enzyme; ACEi: ACE inhibitors; AcPGP: acetyl PGP; ANG II: angiotensin II; ARB: angiotensin II receptor blockers; ARDS: acute respiratory distress syndrome; CKD: chronic kidney disease; COPD: chronic obstructive pulmonary disease; COVID-19: coronavirus disease 2019; ICU: intensive care unit; MMPs: matrix metalloproteinases; MRA: mineralocorticoid receptor antagonists; POP: Prolyloligopeptidase; RAS: renin–angiotensin system; RAASi: renin–angiotensin–aldosterone system inhibitors; SARS-CoV-2: severe acute respiratory syndrome coronavirus 2; STAT3: Signal Transducer And Activator Of Transcription 3; T2DM: type 2 diabetes mellitus; TMPRSS2: transmembrane protease serine 2.
